# Video-Assisted Thoracoscopic Surgery Versus Thoracotomy for Pleural Empyema: Outcomes and Risk Factors in a Retrospective Single-Center Cohort Study

**DOI:** 10.1186/s13019-026-03992-3

**Published:** 2026-04-08

**Authors:** Josef Yayan, Amr Khalil, Marcus Krüger, Christian Biancosino

**Affiliations:** 1 Department of Internal Medicine, Division of Pulmonary, Allergy, and Sleep Medicine, HELIOS Clinic Wuppertal, Witten/Herdecke University, Heusnerstr. 40, Wuppertal, 42283 Germany; 2 Department of Thoracic Surgery, HELIOS Clinic Wuppertal, Witten/Herdecke University, Wuppertal, Germany; 3 Department of Thoracic Surgery, Martha-Maria Hospital Halle-Dölau, Halle, Germany

**Keywords:** Pleural empyema, Surgical outcomes, Postoperative complications, Thoracotomy, Video-assisted thoracoscopic surgery (VATS)

## Abstract

**Background:**

Pleural empyema is a serious complication of pulmonary infections that often requires surgical intervention . This study compared outcomes between video-assisted thoracoscopic Surgery (VATS) and thoracotomy, with particular attention to postoperative complications, intensive care unit (ICU) admission rates, and intraoperative parameters. The aim was to evaluate associations between surgical approach and perioperative outcomes in routine clinical practice.

**Methods:**

We retrospectively reviewed 103 patients who underwent surgical management for pleural empyema at a university thoracic surgery department between December 2019 and May 2024. Nineteen patients (18.4%) underwent VATS, and 84 patients (81.6%) underwent thoracotomy. The surgical approach was determined based on disease stage and intraoperative surgical assessment. Primary outcomes included postoperative complications and ICU admissions, while secondary outcomes included transfusion requirements and operative duration. Ethical approval was obtained prior to study initiation.

**Results:**

Complications occurred significantly less frequently following VATS than after thoracotomy (10.5% vs. 51.2%, *p* = 0.005). ICU admissions were also less common in the VATS group (57.9% vs. 86.9%, *p* = 0.005). No patients in the VATS group required transfusions, whereas 28.6% patients in the thoracotomy group did (*p* = 0.057). Operative time did not differ significantly between the groups (VATS: 99.6 min; thoracotomy: 116.4 min, *p* = 0.181). Intraoperative pathogen detection rates were similar (OR 0.88, 95% CI: 0.301–2.544, *p* = 0.807).

**Conclusions:**

In this retrospective cohort, VATS was associated with lower rates of postoperative complications and ICU admissions compared with thoracotomy in appropriately selected patients. Thoracotomy remains essential indispensable in advanced disease requiring extensive decortication, likely reflecting underlying disease severity rather than the surgical approach itself . Given the retrospective design and imbalance in group sizes , these findings should be interpreted with caution. Prospective multicenter studies are needed to confirm these findings and refine surgical decision-making criteria.

## Introduction

Pleural empyema, characterized by the accumulation of purulent or infected fluid within the pleural cavity, continues to represent a major therapeutic challenge, especially when conservative management fails and surgery becomes necessary. It most often develops as a complication of pneumonia, pulmonary abscesses, or prior thoracic procedures, and in the absence of effective treatment it can lead to substantial morbidity and mortality [1]. Initial therapy typically consists of antimicrobial treatment and chest tube drainage, but advanced stages generally require operative intervention. The two principal surgical strategies—video-assisted thoracoscopic surgery (VATS) and open thoracotomy—have been widely applied, yet the choice between them remains a subject of ongoing discussion in modern thoracic surgery [2].

Over the past decades, VATS has become increasingly favored owing to its minimally invasive nature. Reported advantages include reduced postoperative pain, accelerated convalescence, and a lower rate of perioperative complications compared with thoracotomy [3]. Preservation of chest wall integrity and less impairment of respiratory mechanics make VATS particularly attractive for patients with early-stage disease. Nevertheless, in complex or late-stage empyema, thoracotomy is still often preferred because it provides superior exposure for complete pleural decortication [4].

Evidence from observational studies indicates that thoracotomy is frequently associated with higher morbidity, longer hospitalization, and a greater risk of complications, particularly in individuals with relevant comorbidities [5]. Importantly, the allocation of patients to either VATS or thoracotomy is rarely random; rather, it reflects underlying disease extent, baseline condition, and the treating surgeon’s expertise. This selection bias complicates interpretation of outcome differences, as patients with advanced empyema or compromised health are more likely to undergo thoracotomy [6]. Established risk factors such as older age, tobacco use, chronic lung disease, and poor preoperative functional status are known to adversely influence outcomes irrespective of surgical approach [7]. However, few investigations have systematically examined how these risks interact with the chosen operative technique.

A more refined understanding of patient- and procedure-related factors that contribute to postoperative complications is therefore needed. In particular, analyses that consider demographic characteristics, comorbidity burden, preoperative condition, and intraoperative aspects such as operative duration and blood loss may help identify vulnerable subgroups [8].

Based on this rationale, the present study aimed to explore the association between surgical approach and perioperative outcomes in patients undergoing surgery for pleural empyema. The objective of the study was to compare outcomes between VATS and thoracotomy and to delineate patient characteristics that predispose to postoperative adverse events. By addressing these aspects in a real-world, retrospective cohort, this analysis seeks to contribute additional data to the ongoing discussion regarding the optimal surgical management of pleural empyema [9].

## Materials and methods

### Study design and setting

This retrospective observational study was carried out at the Department of Thoracic Surgery, University Hospital Helios Wuppertal, Germany with the objective of identifying risk factors for adverse outcomes in patients treated for pleural empyema. Outcomes of video-assisted thoracoscopic surgery (VATS) were compared with those of open thoracotomy. Data collection covered the period from December 1, 2019, to May 31, 2024.

### Study population

The study included 103 consecutive adult patients with a confirmed diagnosis of pleural empyema who underwent surgical treatment during the defined study period. Of these, 19 patients (18.4%, 95% CI: 11.0–27.0) underwent VATS and 84 (81.6%, 95% CI: 73.0–89.0) underwent thoracotomy. Inclusion criteria were age ≥ 18 years and a clear clinical or radiological diagnosis of empyema requiring operative intervention. Patients were excluded if they had undergone previous surgical treatment for empyema within the preceding six months or if their clinical records were incomplete. Preoperative empyema stage was retrospectively classified according to the American Thoracic Society/American Association for Thoracic Surgery (ATS/AATS) classification into stage I, II, or III based on imaging findings, intraoperative appearance, and operative reports. Conversions from VATS to thoracotomy were classified and analyzed within the thoracotomy group to reflect clinical practice. Parts of this cohort have been previously analyzed focusing on age-related and sex-specific aspects; the present study addresses a distinct research question concerning surgical approach.

### Data collection

Clinical information was extracted from electronic hospital records. Variables collected included demographic characteristics (age, sex, body mass index), comorbidities (smoking history, hypertension, diabetes mellitus, chronic obstructive pulmonary disease, cardiovascular diseases), and preoperative laboratory parameters such as leukocyte count, C-reactive protein (CRP), and serum creatinine. The American Society of Anesthesiologists (ASA) physical status score was used to stratify patients according to perioperative risk, ranging from ASA I (healthy) to ASA V (moribund). Surgical details such as procedure type (VATS vs. thoracotomy), operative duration, intraoperative blood loss, and transfusion requirements were also recorded. The extent of surgery (pleural debridement alone versus lung decortication) was documented based on operative reports. Continuous variables were summarized as mean values with standard deviation (SD), while categorical variables were presented as absolute numbers and percentages.

### Surgical procedures

The surgical strategy was selected by the operating surgeon according to disease stage and overall patient condition. VATS was performed using a uniportal thoracoscopic technique through a single skin incision of approximately 3–4 cm, without the use of multiple ports or trocars, as previously described in the literature. The procedure aimed at the evacuation of purulent material and pleural debridement, with lung decortication performed when required based on intraoperative findings. Thoracotomy involved a larger incision, providing direct exposure for extensive decortication and debridement when required. All procedures were performed under general anesthesia with single-lung ventilation and were carried out by board-certified thoracic surgeons with at least five years of independent operative experience. The decision-making process inherently carried a degree of selection bias, as more advanced cases were usually assigned to thoracotomy.

### Outcome measures

The main study endpoint was the occurrence of postoperative complications, defined as follows:

- Pneumonia: new infiltrates on chest imaging combined with fever, leukocytosis, or positive cultures.
- Pleural abscess or recurrence: radiologic or microbiological evidence of persistent or recurrent infected effusion requiring intervention.
- Respiratory failure: need for prolonged ventilation (> 48 h) or re-intubation.
- Sepsis: systemic inflammatory response with clinical or microbiological confirmation of infection.

Additional outcomes included admission to an intensive care unit (ICU) and all-cause in-hospital mortality. Admission to the ICU was based on institutional criteria, including the need for postoperative mechanical ventilation, hemodynamic monitoring, or treatment of severe infection. Secondary endpoints were operative time, length of hospital stay, and the requirement for postoperative transfusion or surgical re-intervention within 30 days.

### Statistical analysis

Continuous data were described as means ± SD, and categorical variables as percentages. Between-group comparisons for normally distributed continuous data were performed with independent-samples t-tests, while non-normally distributed variables were assessed using Mann–Whitney U tests. Chi-square or Fisher’s exact tests were applied for categorical data, with odds ratios (OR) and 95% confidence intervals (CI) reported. Multivariable logistic regression was used to evaluate the association between surgical approach and postoperative complications, adjusting for potential confounders including age, comorbidities, ASA score, and empyema stage. A two-tailed p value < 0.05 was considered statistically significant.

### Data protection and confidentiality

All patient information was anonymized before analysis, and data were stored securely in compliance with institutional standards and the General Data Protection Regulation (GDPR). Access to identifiable data was restricted to authorized study personnel only.

## Results

A total of 103 patients who underwent surgical treatment for pleural empyema between December 2019 and May 2024 were included in this study. Of these, 19 patients (18.4%, 95% CI: 11.0–27.0) underwent VATS and 84 patients (81.6%, 95% CI: 73.0–89.0) underwent thoracotomy, including three patients converted from VATS to thoracotomy due to advanced disease stage or incomplete lung re-expansion. These patients were analyzed within the thoracotomy group.

### Baseline characteristics

No significant differences were observed between groups with respect to demographic parameters. The mean age was 56.7 years (95% CI: 50.0–63.4) in the VATS cohort and 59.8 years (95% CI: 55.1–64.5) in the thoracotomy cohort (*p* = 0.559). The proportion of male patients was 63.2% (95% CI: 43.6–81.6) in the VATS group and 72.6% (95% CI: 61.1–83.7) in the thoracotomy group (*p* = 0.410). Comorbidities, including smoking, arterial hypertension, and chronic obstructive pulmonary disease, were similarly distributed between groups (Table 1). According to the ATS/AATS classification, patients treated with VATS predominantly presented with stage II empyema, whereas stage III empyema was more frequently observed in the thoracotomy group (Table 1).

**Table 1:** Comparison of demographic, clinical, and surgical characteristics between patients undergoing video-assisted thoracoscopic surgery (VATS, *n* = 19) and thoracotomy (*n* = 84) for pleural empyema

Variable	VATS (*n* = 19) (%)	Thoracotomy (*n* = 84) (%)	Odds ratio (95% CI)	*P* value
Male	12 (63.2)	61 (72.6)	0.65 (0.23–1.84)	0.41
Age (years, mean ± SD)	56.7 ± 21.3	59.8 ± 15.6	—	0.559
Smoking	7 (36.8)	35 (41.7)	0.82 (0.29–2.28)	0.70
Anticoagulant therapy	4 (21.1)	13 (15.5)	1.46 (0.42–5.10)	0.56
ASA I	3 (15.8)	5 (6.0)	2.96 (0.64–13.67)	0.164
ASA II	5 (26.3)	23 (27.4)	0.95 (0.31–2.93)	0.92
ASA III	11 (57.9)	55 (65.5)	0.73 (0.26–2.0)	0.535
Right lung empyema	12 (63.2)	46 (54.8)	1.42 (0.51–4.0)	0.506
Left lung empyema	7 (36.8)	38 (45.2)	0.71 (0.25–1.97)	0.506
Postoperative complications	2 (10.5)	43 (51.2)	0.11 (0.02–0.48)	**0.005**
Intensive Care Unit (ICU)	11 (57.9)	73 (86.9)	0.21 (0.07–0.63)	**0.005**
Blood transfusion	0	24 (28.6)	0.06 (0.00–1.09)	0.057
Intraoperative pathogen detection	6 (31.6)	29 (34.5)	0.88 (0.30–2.54)	0.807
Surgery duration (min, mean ± SD)	99.6 ± 50.6	116.4 ± 32.1	—	0.181
Arterial hypertension	2 (10.5)	26 (31.0)	0.26 (0.06–1.22)	0.088
Chronic kidney insufficiency	2 (10.5)	5 (6.0)	1.86 (0.33–10.40)	0.480
Hypertensive heart disease	4 (21.1)	11 (13.1)	1.77 (0.50–6.32)	0.379
Diabetes mellitus	3 (15.8)	10 (11.9)	1.39 (0.34–5.62)	0.646
Obesity	1 (5.3)	4 (4.8)	1.11 (0.12–10.54)	0.927
Chronic obstructive pulmonary disease (COPD)	1 (5.3)	10 (11.9)	0.41 (0.05–3.42)	0.411
Coronary heart disease	3 (15.8)	5 (6.0)	2.96 (0.64–13.67)	0.164

Data are presented as mean ± standard deviation (SD) for continuous variables and as number (percentage) for categorical variables. Odds ratios (OR) with 95% confidence intervals (CI) and two-sided p values are reported for group comparisons. Statistical analyses were performed using independent-samples t test or Mann–Whitney U test for continuous variables and logistic regression or Fisher’s exact test, as appropriate, for categorical variables. ICU = intensive care unit; VATS = video-assisted thoracoscopic surgery. ASA: American Society of Anesthesiologists physical status classification. significant *P* values are in bold

### Postoperative complications

**Fig. 1:**
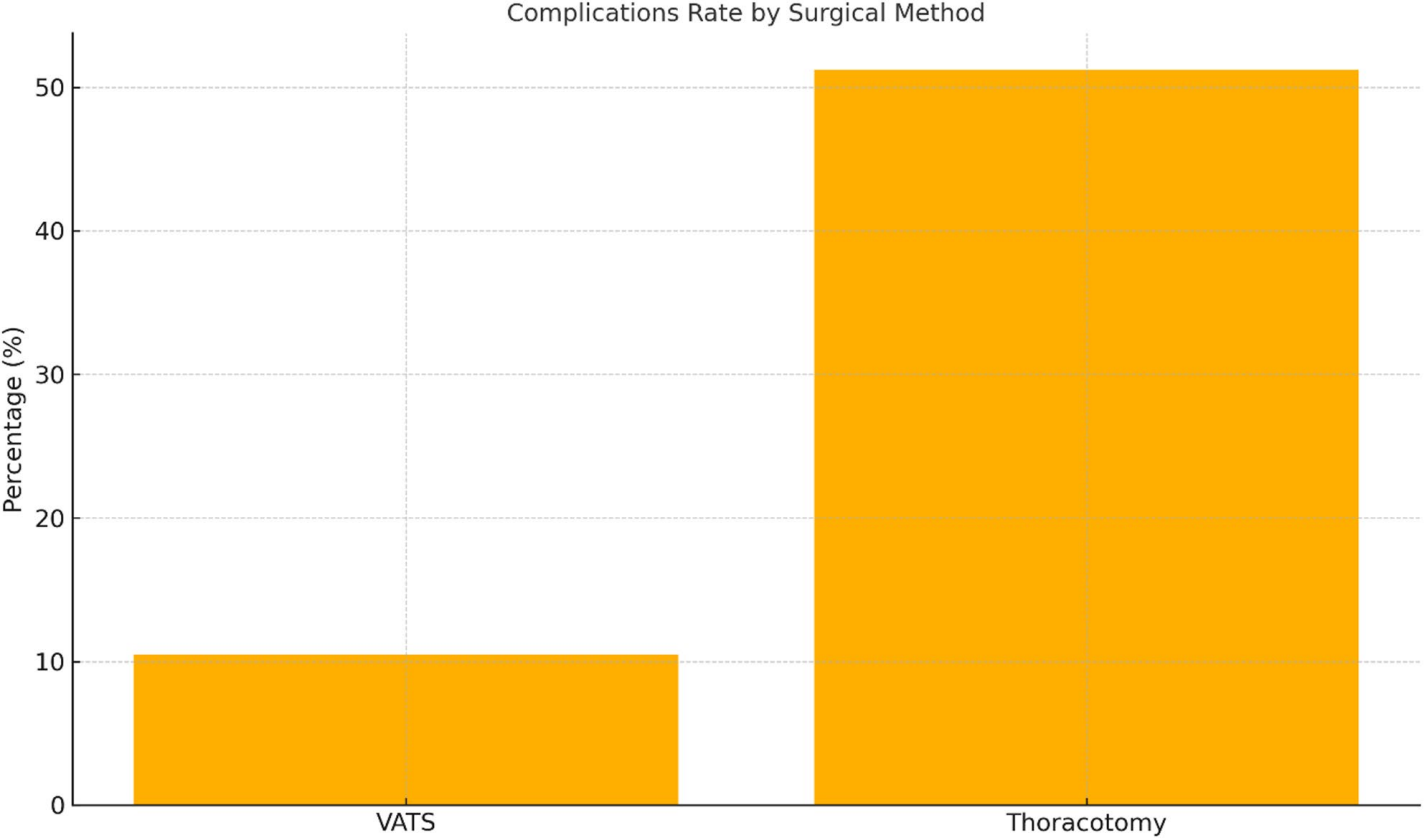
Complication Rate by Surgical Method. This bar chart compares the postoperative complication rates between patients undergoing video-assisted thoracoscopic surgery (VATS) and those treated with thoracotomy for pleural empyema. Postoperative complications occurred less frequently in patients treated with VATS (10.5%) than in those undergoing thoracotomy (51.2%). This difference was statistically significant (*p* = 0.005)

Postoperative complications occurred in 2 of 19 patients treated with VATS (10.5%, 95% CI: 1.9–27.5%) compared with 43 of 84 patients undergoing thoracotomy (51.2%, 95% CI: 41.1–61.3%). This corresponded to an odds ratio (OR) of 0.11 (95% CI: 0.02–0.48, *p* = 0.005). After adjustment for age, ASA classification, and comorbidity burden, and empyema stage, VATS remained independently associated with a lower risk of postoperative complications (adjusted OR 0.14, 95% CI: 0.03–0.61, *p* = 0.009) (Fig. 1). Higher complication rates were observed in patients with stage III empyema, irrespective of surgical approach.

### ICU admission

**Fig. 2:**
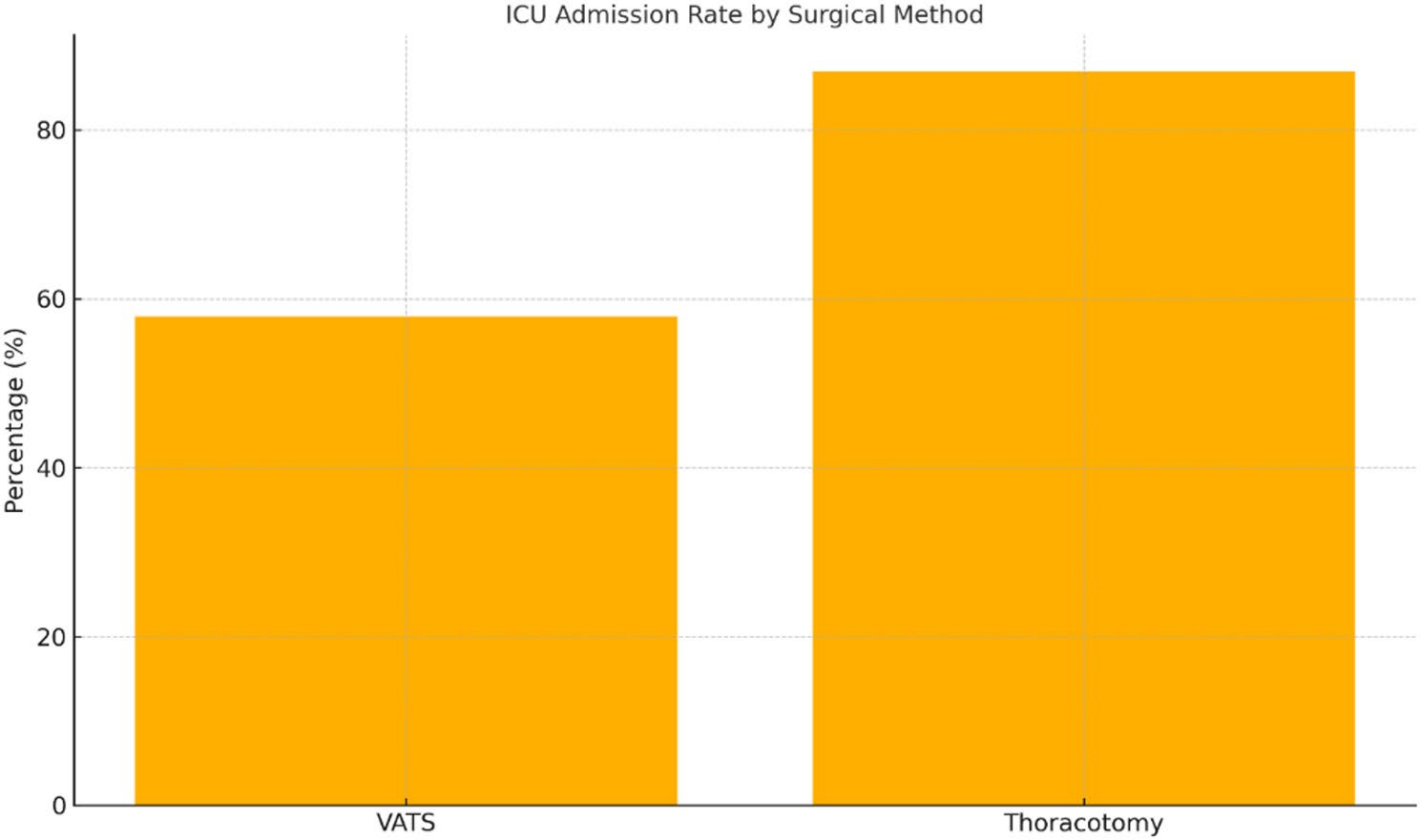
Intensive care unit (ICU) admission rates after surgical treatment of pleural empyema. Admission to the ICU was required less frequently in patients treated with video-assisted thoracoscopic surgery (VATS) than in those treated with thoracotomy (57.9% vs. 86.9%). This difference was statistically significant (*p* = 0.005)

Admission to the intensive care unit (ICU) was required in 11 of 19 patients in the VATS group (57.9%, 95% CI: 36.5–77.6%) and in 73 of 84 patients in the thoracotomy group (86.9%, 95% CI: 77.3–96.4%). The unadjusted OR for ICU admission was 0.19 (95% CI: 0.06–0.60, *p* = 0.005). In multivariable analysis, VATS continued to show a protective effect (adjusted OR 0.22, 95% CI: 0.07–0.71, *p* = 0.012) (Fig. 2). ICU admission was more frequent in patients with advanced empyema stages and in those requiring lung decortication.

### Intraoperative findings

The rate of intraoperative pathogen detection was similar between groups, with rates of 31.6% for VATS and 34.5% for thoracotomy (OR 0.88, 95% CI: 0.30–2.54, *p* = 0.807) (Table 1).

### Extent of surgery

Pleural debridement alone was performed more frequently in the VATS group, whereas lung decortication was predominantly required in patients undergoing thoracotomy, reflecting more advanced disease stages (Table 1).

### Blood transfusions

**Fig. 3:**
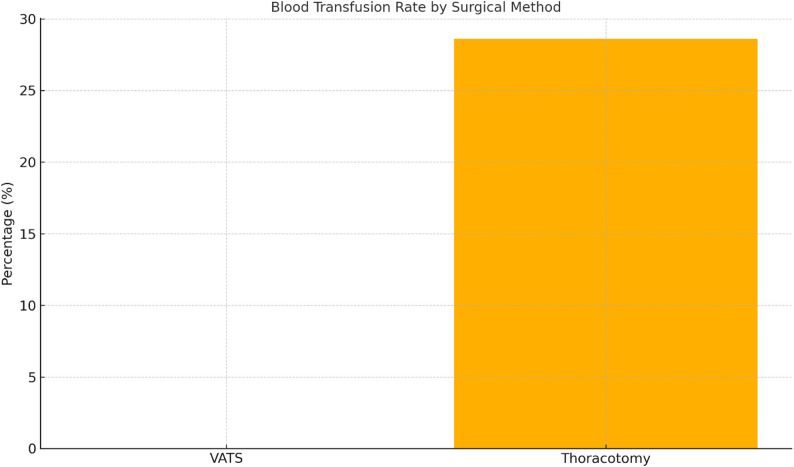
Requirement of intraoperative or postoperative blood transfusions by surgical method. None of the patients in the video-assisted thoracoscopic surgery (VATS) group required a transfusion, whereas 28.6% of patients in the thoracotomy group did. This difference did not reach statistical significance but indicated a trend toward lower transfusion requirements in the VATS group (*p* = 0.057)

None of the VATS patients required transfusion, compared with 24 of 84 patients in the thoracotomy group (28.6%, 95% CI: 19.1–39.6%). Although this difference narrowly missed statistical significance (*p* = 0.057), the trend suggested a clinically meaningful reduction in transfusion requirements with VATS (Fig. 3).

### Operative time

The mean operative duration was 99.6 min (95% CI: 85.2–114.0) for VATS and 116.4 min (95% CI: 106.0–126.9) for thoracotomy. The difference was not statistically significant (*p* = 0.181).

### Summary

Taken together, patients undergoing VATS experienced significantly fewer complications and ICU admissions than those treated by thoracotomy. These differences were closely related to empyema stage and the extent of surgical intervention, underscoring the importance of disease severity and patient selection when interpreting outcome differences between surgical approaches.

## Discussion

This study provides a detailed comparison of outcomes between video-assisted thoracoscopic surgery (VATS) and thoracotomy in patients with pleural empyema, with emphasis on postoperative complications and related risk factors. Rather than demonstrating causality, the present analysis identifies associations between surgical approach and short-term outcomes within a retrospective cohort. Patients undergoing VATS demonstrated lower complication rates and fewer ICU admissions compared with those treated by thoracotomy.

The markedly lower rate of complications in the VATS cohort (10.5%) compared with the thoracotomy group (51.2%) is consistent with earlier reports highlighting the benefits of a minimally invasive approach [10, 11]. However, these differences must be interpreted in the context of disease severity. Reduced surgical trauma to the chest wall and a lower susceptibility to infection likely contribute to these improved outcomes. Importantly, the complications observed in this study—including pneumonia, respiratory failure, and sepsis—were more common in patients with advanced empyema stages, suggesting that disease burden rather than surgical approach alone may drive postoperative morbidity. This observation is in line with previous investigations that reported shorter hospital stays, fewer pulmonary sequelae, and faster recovery following thoracoscopic surgery in appropriately selected patients [12].

Nevertheless, thoracotomy continues to play a central role in patients with advanced empyema requiring extensive pleural decortication. The higher complication rate seen in the thoracotomy group most likely reflects the more severe disease burden in this subset, underscoring the inherent selection bias that arises when sicker patients are preferentially directed toward open procedures. This mirrors everyday surgical practice, where clinical judgment and disease stage are decisive in choosing the operative approach [13]. In the present study, patients requiring conversion from VATS to thoracotomy were analyzed within the thoracotomy group, which may have further contributed to outcome differences between groups.

Another key finding was the significantly lower need for ICU care in the VATS cohort (57.9%) compared with the thoracotomy cohort (86.9%). This aligns with the established advantages of VATS in reducing postoperative pain, facilitating earlier mobilization, and limiting the need for prolonged intensive monitoring [14]. It should be noted, however, that ICU admission rates may also reflect institutional practice patterns and the higher prevalence of advanced empyema stages among thoracotomy patients, rather than postoperative morbidity alone. Beyond individual patient benefits, this reduction in ICU utilization suggests a potential healthcare system advantage through decreased resource consumption.

Blood transfusions were required exclusively in the thoracotomy group (28.6%), whereas no patient in the VATS group needed transfusion support. This observation highlights the hemostatic benefit of minimally invasive techniques, in line with prior studies demonstrating that reduced intraoperative blood loss enhances recovery and lowers postoperative infection risk [15]. At the same time, the greater need for transfusion in the thoracotomy group likely reflects the higher frequency of extensive decortication procedures in advanced empyema.

The average duration of surgery was similar between groups (99.6 vs. 116.4 min, *p* = 0.181). Prior research likewise suggests that VATS does not necessarily shorten operative time, especially in advanced disease [16]. Instead, the benefits of VATS appear to be derived primarily from less invasive access rather than shorter procedure length, with measurable improvements in perioperative outcomes and ICU requirements.

Microbiological findings showed no significant difference in pathogen detection between the two groups. This suggests that outcomes are less influenced by intraoperative microbiological yield and more by patient-related factors, disease stage, and surgical extent.

Taken together, these findings emphasize the importance of careful patient selection. While VATS was associated with more favorable short-term outcomes, it may not be feasible or appropriate in patients with advanced fibrinous empyema or those requiring extensive decortication. Risk stratification incorporating age, comorbidity burden, and empyema stage is essential to optimize individualized surgical decision-making [17].

In summary, this retrospective single-center analysis demonstrates an association between VATS and lower rates of postoperative complications and ICU admissions in selected patients with pleural empyema. Thoracotomy, however, remains indispensable for complex and advanced cases requiring extensive decortication. Given the retrospective design, group size imbalance, and potential selection bias, these findings should be interpreted with caution. Further prospective, multicenter, and ideally randomized studies are needed to refine patient selection criteria, reduce selection bias, and confirm the role of VATS in broader clinical populations [18]. Therefore, this study should be interpreted as hypothesis generating rather than practice-changing.

### Limitations

This study has several noteworthy limitations. First, its retrospective and single-center nature limits the ability to establish causal relationships and introduces the risk of selection bias. Patients with more advanced empyema were more frequently assigned to thoracotomy, which may partly account for the higher complication rates observed in this group. Although empyema stage was considered in the analysis, residual confounding related to disease severity cannot be excluded. Second, the sample size was modest, particularly within the VATS cohort (*n* = 19), which reduces statistical power and limits the generalizability of the findings. The low number of events in the VATS group also restricts the robustness of adjusted analyses and warrants cautious interpretation of effect estimates. Third, although all operations were performed by board-certified thoracic surgeons, differences in individual surgical technique and intraoperative decision-making could have influenced perioperative outcomes. Fourth, conversions from VATS to thoracotomy were analyzed as part of the thoracotomy group; while this reflects routine clinical practice, it may have introduced heterogeneity and affected comparability between groups. In addition, the inclusion of converted cases in the thoracotomy group may have amplified outcome differences between approaches. Fifth, the analysis focused exclusively on short-term perioperative outcomes and did not assess longer-term endpoints such as recurrence, functional recovery, or patient-reported quality of life, which are essential for a comprehensive evaluation of surgical strategies. Finally, although intraoperative microbiological detection was documented, the potential influence of specific pathogens on outcomes was not examined. Similarly, ICU admission rates may have been influenced by institutional practice patterns rather than postoperative morbidity alone. Future research should address these limitations through larger, multicenter prospective studies, ideally with randomized designs, to confirm our observations and to refine patient selection criteria for VATS and thoracotomy in the management of pleural empyema.

## Conclusion

Our findings indicate that video-assisted thoracoscopic surgery (VATS) was associated with more favorable short-term outcomes compared with thoracotomy in the surgical management of pleural empyema, including significantly lower complication rates, fewer ICU admissions, and the absence of transfusion requirements. These results underscore the value of minimally invasive techniques in enhancing postoperative recovery and reducing the overall healthcare burden. Nevertheless, thoracotomy remains essential for patients with advanced or complex disease who require extensive decortication. The observed differences between surgical approaches likely reflect underlying disease severity and patient selection rather than the operative technique alone. When technically feasible and in appropriately selected patients, VATS may represent a preferred surgical approach. Further prospective and randomized trials are needed to establish robust selection criteria and to assess long-term outcomes, including recurrence, functional recovery, and quality of life.

## Data Availability

All data are included in the manuscript.

## Declarations

### Artificial intelligence usage

No generative artificial intelligence (AI) tools were used in the research design, data analysis, or writing of this manuscript. All content was generated by the authors without the assistance of AI-based technologies.

## References

[1] Redden MD, Chin TY, van Driel ML. Surgical versus non-surgical management for pleural empyema. Cochrane Database Syst Rev. 2017;3(3):CD010651. 10.1002/14651858.CD010651.pub2. PMID: 28304084; PMCID: PMC6464687. 28304084 10.1002/14651858.CD010651.pub2 PMC6464687

[2] Chen D, Mao R, Kadeer X, Sun W, Zhu E, Peng Q, Chen C. Video-assisted thoracic surgery is an optimal alternative to conventional thoracotomy for reoperations for ipsilateral pulmonary lesions. Thorac Cancer. 2018;9(11):1421–8. 10.1111/1759-7714.12854. PMID: 30152592; PMCID: PMC6209788. 30152592 10.1111/1759-7714.12854 PMC6209788

[3] Mazzei M, Abbas AE. Why comprehensive adoption of robotic assisted thoracic surgery is ideal for both simple and complex lung resections. J Thorac Dis. 2020;12(2):70–81. 10.21037/jtd.2020.01.22. PMID: 32190356; PMCID: PMC7061192. 32190356 10.21037/jtd.2020.01.22 PMC7061192

[4] Subotic D, Lardinois D, Hojski A. Minimally invasive thoracic surgery for empyema. Breathe (Sheff). 2018;14(4):302–10. 10.1183/20734735.025718. PMID: 30519296; PMCID: PMC6269178. 30519296 10.1183/20734735.025718 PMC6269178

[5] Sengupta S. Post-operative pulmonary complications after thoracotomy. Indian J Anaesth. 2015;59(9):618–26. 10.4103/0019-5049.165852. PMID: 26556921; PMCID: PMC4613409. 26556921 10.4103/0019-5049.165852 PMC4613409

[6] Mohajerzadeh L, Lotfollahzadeh S, Vosoughi A, Harirforoosh I, Parsay S, Amirifar H, Farahbakhsh N, Atqiaee K. Thoracotomy versus video-assisted thoracoscopy in pediatric empyema. Korean J Thorac Cardiovasc Surg. 2019;52(3):125–30. PMID: 31236371; PMCID: PMC6559187. 31236371 10.5090/kjtcs.2019.52.3.125 PMC6559187

[7] Yang J, Xia Y, Yang Y, Ni ZZ, He WX, Wang HF, Xu XX, Yang YL, Fei K, Jiang GN. Risk factors for major adverse events of video-assisted thoracic surgery lobectomy for lung cancer. Int J Med Sci. 2014;11(9):863–9. 10.7150/ijms.8912. PMID: 25013365; PMCID: PMC4081307. 25013365 10.7150/ijms.8912 PMC4081307

[8] Al-Githmi IS, Alotaibi A, Habeebullah A, Bajunaid W, Jar S, Alharbi NA, Aziz H. Postoperative pulmonary complications in patients undergoing elective thoracotomy versus thoracoscopic surgeries. Cureus. 2023;15(9):e45367. 10.7759/cureus.45367. PMID: 37849610; PMCID: PMC10578611. 37849610 10.7759/cureus.45367 PMC10578611

[9] van Middendorp LB, Franssen S, Gillissen S, Maessen JG, Hulsewé KWE, Vissers YLJ, de Loos ER. Uniportal video-assisted thoracoscopy is a safe approach in patients with empyema requiring surgery. J Thorac Dis. 2020;12(4):1460–6. 10.21037/jtd.2020.02.29. PMID: 32395283; PMCID: PMC7212173. 32395283 10.21037/jtd.2020.02.29 PMC7212173

[10] Reichert M, Pösentrup B, Hecker A, Schneck E, Pons-Kühnemann J, Augustin F, Padberg W, Öfner D, Bodner J. Thoracotomy versus video-assisted thoracoscopic surgery (VATS) in stage III empyema: an analysis of 217 consecutive patients. Surg Endosc*.* 2018;32(6):2664–2675. 10.1007/s00464-017-5961-7. PMID: 29218675. 10.1007/s00464-017-5961-7 29218675

[11] Bertolaccini L, Fornaro G, Ciani O, Prisciandaro E, Crisci R, Tarricone R, Spaggiari L, VATS Group. The impact of surgical experience in VATS lobectomy on conversion and patient quality of life: results from a comprehensive national video-assisted thoracic surgical database. Cancers (Basel). 2023;15(2):410. 10.3390/cancers15020410. PMID: 36672359; PMCID: PMC9857299. 36672359 10.3390/cancers15020410 PMC9857299

[12] Shebl E, Paul M. Parapneumonic pleural effusions and empyema thoracis. In: *StatPearls* [Internet]. Treasure Island (FL): StatPearls Publishing; 2024 Jan–. Available from: https://www.ncbi.nlm.nih.gov/books/NBK534297/30485002

[13] Scarci M, Abah U, Solli P, Page A, Waller D, van Schil P, Melfi F, Schmid RA, Athanassiadi K, Sousa Uva M, Cardillo G. EACTS expert consensus statement for surgical management of pleural empyema. Eur J Cardiothorac Surg. 2015;48(5):642–653. 10.1093/ejcts/ezv272. PMID: 26254467. 10.1093/ejcts/ezv272 26254467

[14] Miskovic A, Lumb AB. Postoperative pulmonary complications. Br J Anaesth. 2017;118(3):317–334. 10.1093/bja/aex002. PMID: 28186222. 10.1093/bja/aex002 28186222

[15] Yao L, Wang W. Effect of intraoperative blood loss on postoperative pulmonary complications in patients undergoing video-assisted thoracoscopic surgery. Turk Gogus Kalp Damar Cerrahisi Derg. 2021;29(3):347–53. 10.5606/tgkdc.dergisi.2021.20657. PMID: 34589253; PMCID: PMC8462118. 34589253 10.5606/tgkdc.dergisi.2021.20657 PMC8462118

[16] Steen K, Sørensen J, Christensen M, Petersen RH, Naidu B, Bendixen M, Rahman NM, Laursen CB, Christensen TD. Comparison of video-assisted thoracoscopic surgery and thoracotomy for treatment of pleural infection stage II and III: a literature review. J Thorac Dis. 2023;15(11):6323–32. 10.21037/jtd-23-928. PMID: 38090316; PMCID: PMC10713287. 38090316 10.21037/jtd-23-928 PMC10713287

[17] Ricciardi S, Giovanniello D, Carleo F, Di Martino M, Jaus MO, Mantovani S, Treggiari S, Tritapepe L, Cardillo G. Which surgery for stage II–III empyema patients? Observational single-center cohort study of 719 consecutive patients. J Clin Med. 2022;12(1):136. 10.3390/jcm12010136. PMID: 36614937; PMCID: PMC9821231. 36614937 10.3390/jcm12010136 PMC9821231

[18] Wozniak CJ, Paull DE, Moezzi JE, Scott RP, Anstadt MP, York VV, Little AG. Choice of first intervention is related to outcomes in the management of empyema. *Ann Thorac Surg.* 2009;87(5):1525–1530. 10.1016/j.athoracsur.2009.01.028. PMID: 19379898. 10.1016/j.athoracsur.2009.01.028 19379898

